# Monomicrobial Necrotizing Fasciitis of the Breast: A Case Managed by Partial Mastectomy and Hydrogel Dressing

**DOI:** 10.7759/cureus.17891

**Published:** 2021-09-11

**Authors:** Mohammed Mersil, Mohammed Marzouk, Hamad Labeeb

**Affiliations:** 1 Department of General Surgery, Mubarak Alkabeer Hospital, Aljabriya, KWT; 2 Department of General Surgery, Al-Adan Hospital, Hadiya, KWT

**Keywords:** necrotizing fasciitis, breast, hypertension, obesity, partial mastectomy, streptococcus pyogenes, monomicrobial

## Abstract

Necrotizing fasciitis (NF) is a rare life-threatening bacterial infection, which can be monomicrobial or polymicrobial, involving the fascia and eventually leading to necrosis. The course of the disease is rapidly progressive and can be misdiagnosed as an abscess or cellulitis. The disease requires more attention with respect to early diagnosis and treatment as it has a high mortality rate. In this report, we present the case of a 60-year-old female, who was a known case of hypertension and type 2 diabetes mellitus (T2DM). The patient presented to the emergency department on the 21st of October 2020, complaining of left breast pain for 10 days, which was associated with fever and nausea. On physical examination, the left breast was swollen and tender to palpation. There was a single patch of inflamed skin measuring 1 x 1 cm with greenish discoloration in the inframammary fold. Ultrasound of the breast showed a patch of focal mastitis with edema seen at 4-8 o’clock with no underlying fluid collection. She was admitted as a case of left breast abscess and was started on antibiotics. Despite the antibiotic therapy, the patient was still febrile and developed two more inflammatory and necrotic patches with no discharge. The patient underwent urgent surgical debridement of the necrotic tissues, leaving the wound packed for postoperative dressing. The patient stayed in isolation for a total of 25 days as she was found to be positive for severe acute respiratory syndrome coronavirus 2 (SARS-CoV-2). The surgical wound was closed, and the patient was discharged. Early diagnosis and management of NF is the key to saving the patient's life and improving outcomes.

## Introduction

Necrotizing fasciitis (NF), also known as flesh-eating disease, is a rare life-threatening bacterial infection, which can be mono or polymicrobial, involving the fascia and leading to necrosis [1]. The fascia is defined as a sheath or a sheet of aggregative connective tissue where it attaches, encloses, and separates muscles and other internal organs [2]. NF is associated with different signs and symptoms and is classified based on early and late presentations [1]. The early symptoms that may develop within hours to days include high-grade fever, flu-like symptoms, severe tenderness, swelling and redness to the affected area, diarrhea, vomiting, and dark blisters. As the symptoms are nonspecific, up to 85% of NF patients are misdiagnosed. NF tends to be often confused with cellulitis and other superficial skin infections.

NF can also be classified according to its microbiology into monomicrobial or polymicrobial types [1]. Polymicrobial NF is more common and entails a mixture of aerobic and anaerobic organisms and is typically found in the perineum and the trunk [1,3]. Polymicrobial NF often occurs in immunocompromised patients such as those with diabetes or renal failures. On the other hand, monomicrobial NF is less common, affecting healthy individuals, and often occurs in the extremities [4]. Breast NF is a very rare entity and is commonly confused with cellulitis or mastitis [5]. Common risk factors include alcoholic liver disease, diabetes, surgical wounds, and biopsies [5]. We report a case of a diabetic female patient who developed breast NF while being infected with severe acute respiratory syndrome coronavirus 2 (SARS-CoV-2).

## Case presentation

**Figure 1:**
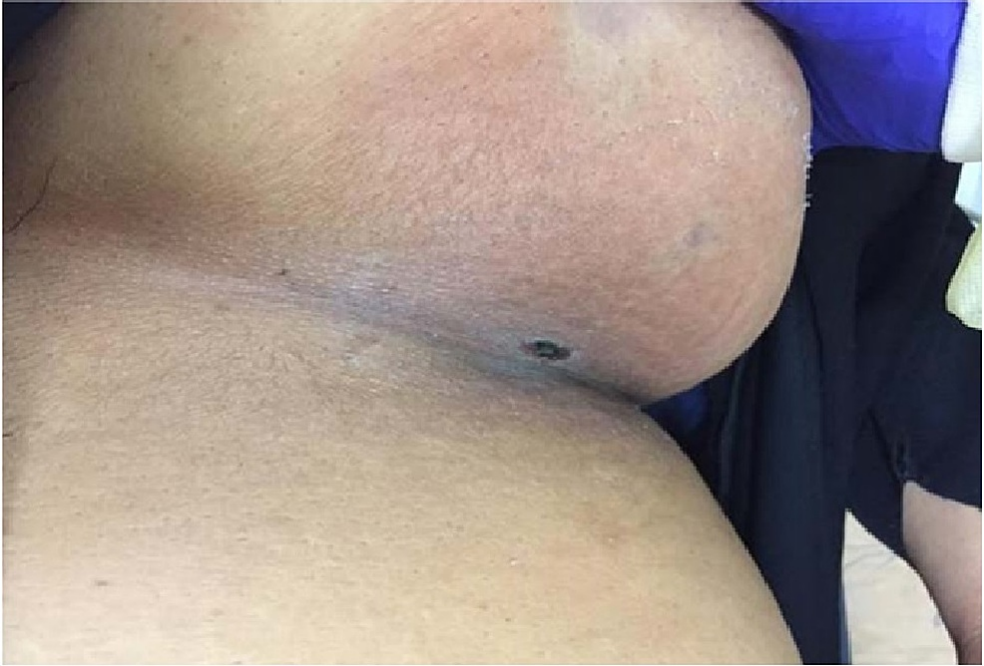
Left breast showing discoloration at admission

Our patient was a 60-year-old obese female who was known to have hypertension and type 2 diabetes mellitus (T2DM). She contracted coronavirus disease 2019 (COVID-19) on the 19th of October 2020. She presented to the Emergency Department on the 21st of October 2019 with a 10-day history of left breast pain. The pain was localized in the lower aspect of the left breast. It was sharp in nature, progressive, and relieved partially by analgesia. The breast pain was associated with high-grade fever, nausea, and swelling. Upon further questioning, it was found that there was neither discharge nor nipple inversion. On examination, the left breast was swollen with green-to-black discoloration and measuring around 1 x 1 cm in the infra-mammary fold (Figure 1).

On palpation of the breast, it was tender and indurated, with palpable left axillary lymph nodes. The patient's temperature was 38.4° C, heart rate was 99/minute, blood pressure was 102/58 mmHg, and SPO_2_ was 99% on room air. Laboratory investigations are summarized in Table 1.

**Table 1:** Laboratory investigation on admission WBC: white blood cells; Hb: hemoglobin

Test	Result	Unit	Reference
WBC	11.1	x 10^9^/L	3.98-10.04
Hb	120	g/L	112-157
Platelet	153	x 10^9^/L	140-400
D-dimer assay	605.48	ng/mL DDU	<270
Creatinine S/P	79	umol/L	44-80
Procalcitonin	1.07	ng/mL	0.02-0.15
C-reactive protein	320.64	mg/L	0-8

**Figure 2:**
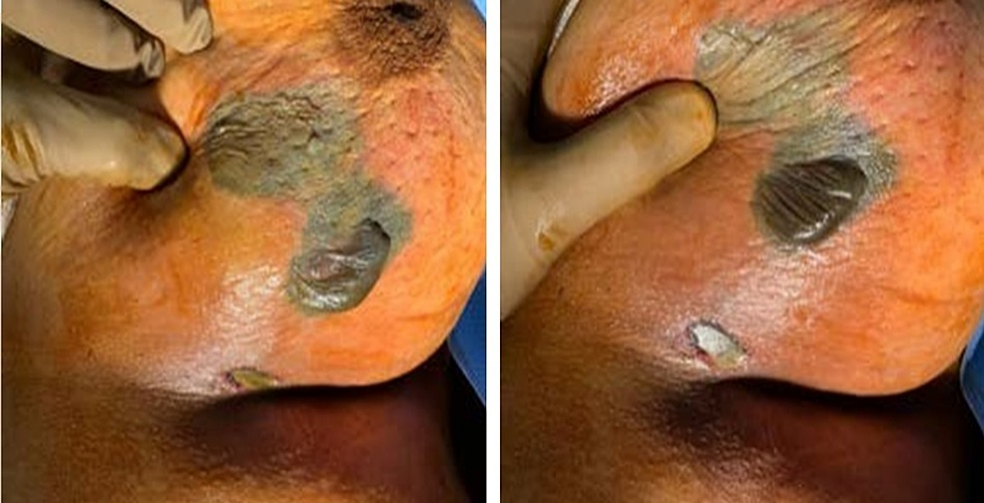
Necrotic tissue seen in the left breast in the operating theatre

Afterward, the patient was sent for ultrasound imaging, which showed a patch of focal mastitis with edema seen at 4-8 o’clock; no underlying localized collection could be seen at the time of examination, but there was thickening of the skin up to 7 mm and multiple enlarged left axillary lymph nodes with the largest being 2 x 0.9 cm in size. As a result, a provisional diagnosis of breast abscess was made due to the high fever and localized inflammation, and the patient was admitted to start on intravenous (IV) antibiotics. On the next day, the patient was still feverish, and the pain had increased. On examination of the left breast, we noticed two necrotic patches with blisters (Figure 2).

**Figure 3:**
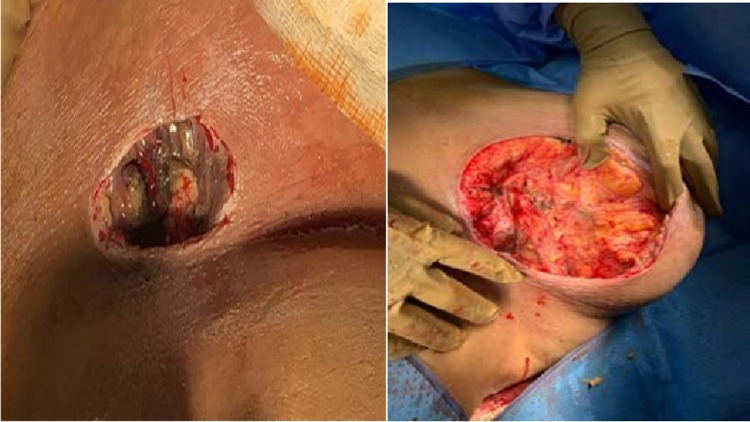
Left: the wound after the excision of the ulcer. Right: necrotic tissues reaching the healthy tissues

Due to the fast development of the necrotic tissues, the patient was offered urgent surgical debridement under general anesthesia. Full surgical debridement was done. Intraoperative findings included large amounts of pus, which was evacuated from the inframammary necrotic ulcer and necrotizing tissue reaching the fascia. An extensive debridement was done involving the skin and subcutaneous layer till reaching the healthy fascia, and the dressing was done without closing the surgical wound (Figure 3).

**Figure 4:**
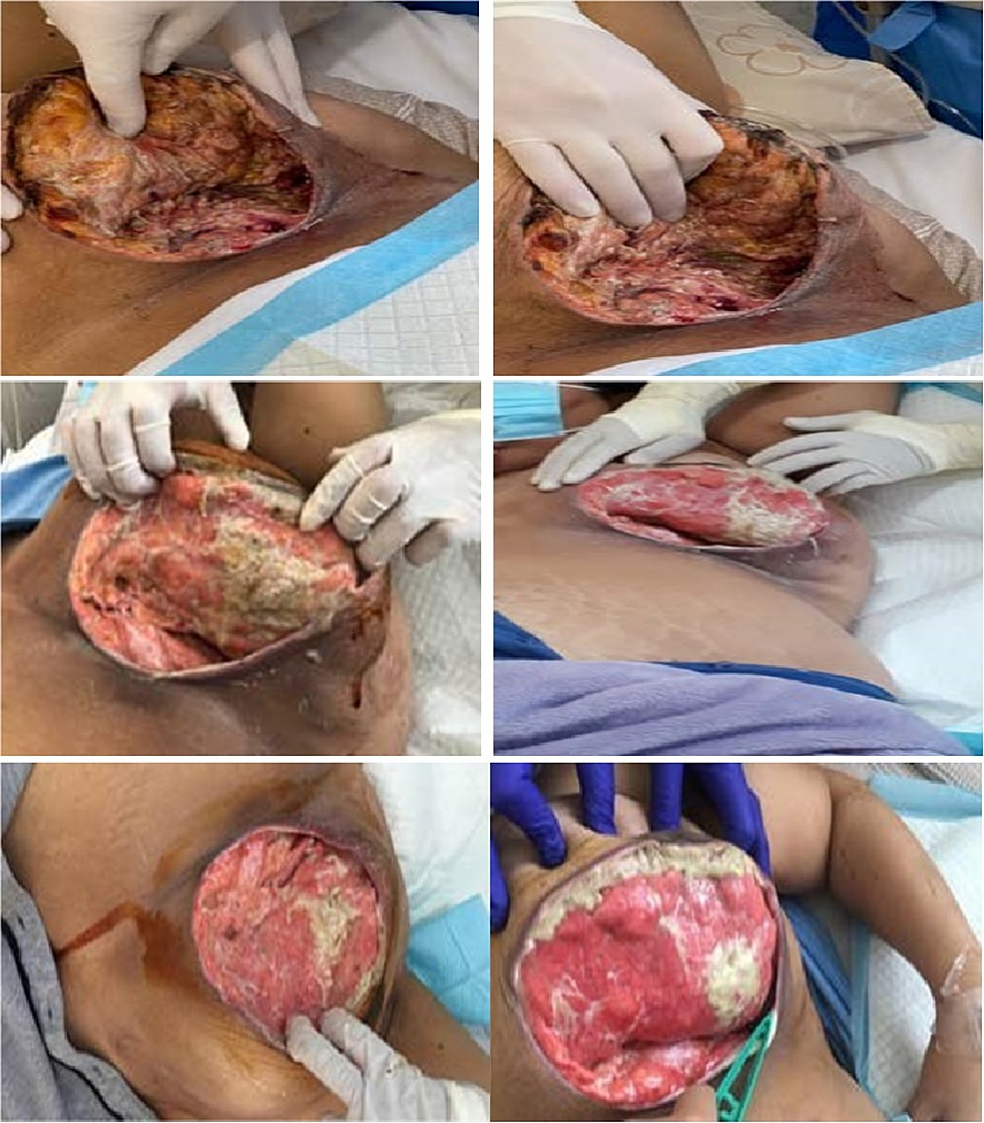
Photographs of the breast showing different stages of the improvement and healing of the wound

A total of three specimens of breast tissue were taken and sent for histopathology, culture, and sensitivity, and SARS-CoV-2 test, and a pus swab was sent for culture and sensitivity. After surgical debridement, the patient was isolated as she was SARS-CoV-2-positive. She underwent multiple bedside surgical debridement, and we used Neogel, which is a range of hydrogel dressings that facilitates chemical debridement of the wound (Figure 4).

**Figure 5:**
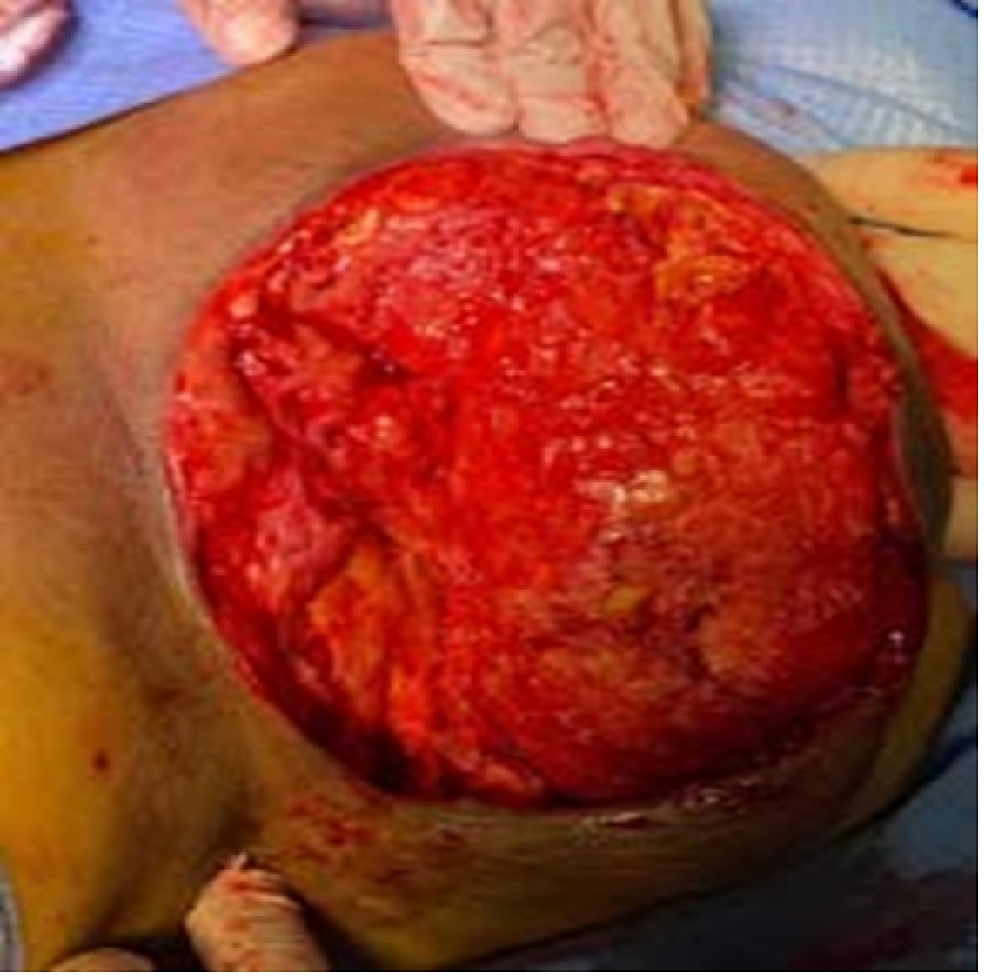
Photograph of the breast during the secondary debridement

The breast tissue histopathology reported necrotic fibrofatty tissue with calcification and severe inflammation. The breast as well as the pus culture and sensitivity reported an isolated *Streptococcus anginosus* growth, which was sensitive to ampicillin and penicillin, and the breast tissue for SARS-CoV-2 returned negative. Therefore, the patient was started on ceftriaxone and ampicillin antibiotics for 14 days and was kept in isolation for a total of 25 days until a SARS-CoV-2 swab returned negative. On the 16th of November 2020, the patient underwent a second debridement in the operation theatre under general anaesthesia. Debridement of the edges was done until reaching healthy tissue. Dressing of the surgical wound without closing can be seen in Figure 5.

**Figure 6:**
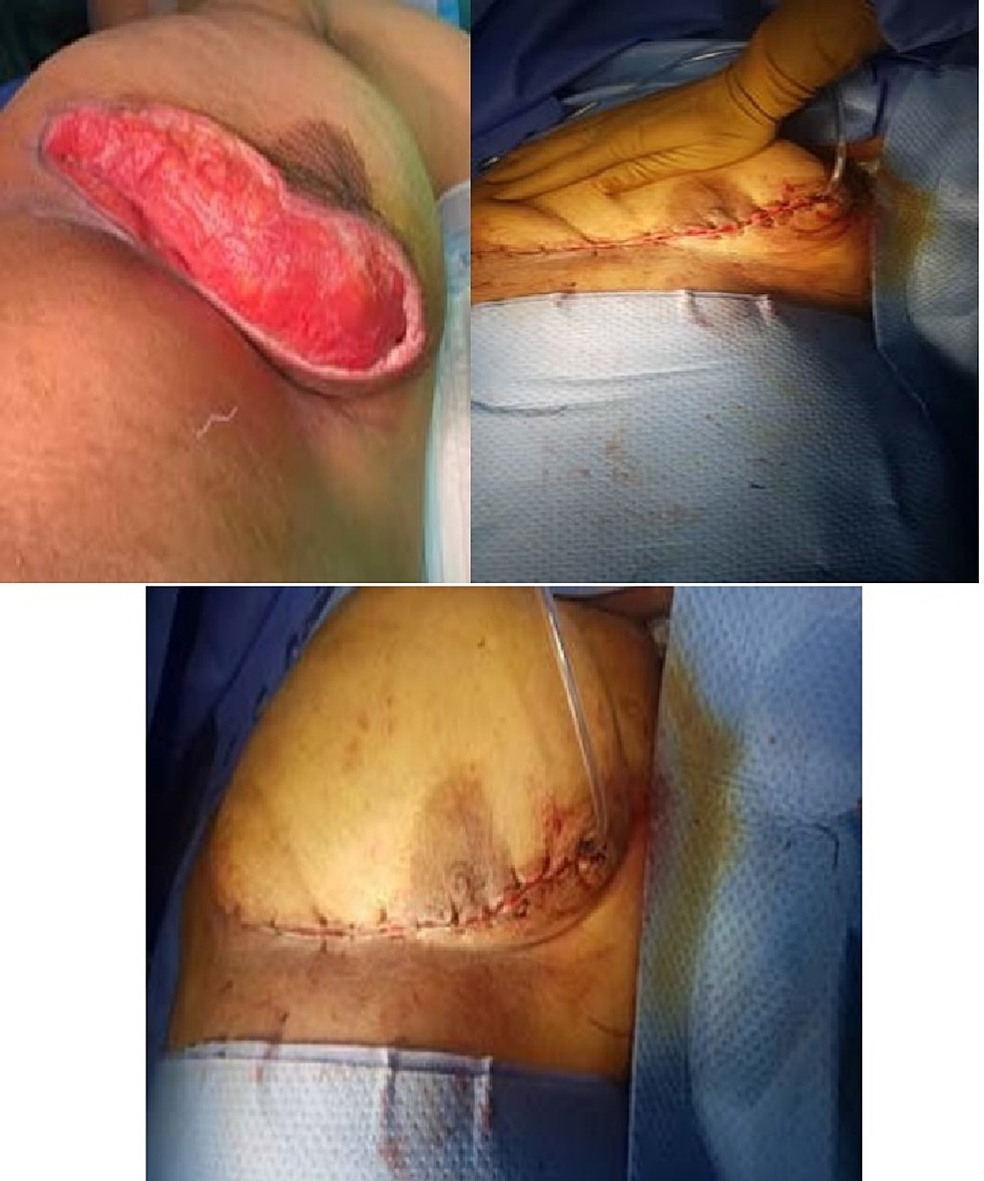
Photographs of the breast after the secondary closure of the wound

The plastic surgery team recommended negative pressure therapy (VAC therapy) and delayed secondary closure. The VAC therapy was applied for five days and then it was stopped. On the 26th of November 2020, the surgical wound was closed by secondary suturing in the main operating theatre under general anaesthesia (Figure 6).

**Figure 7:**
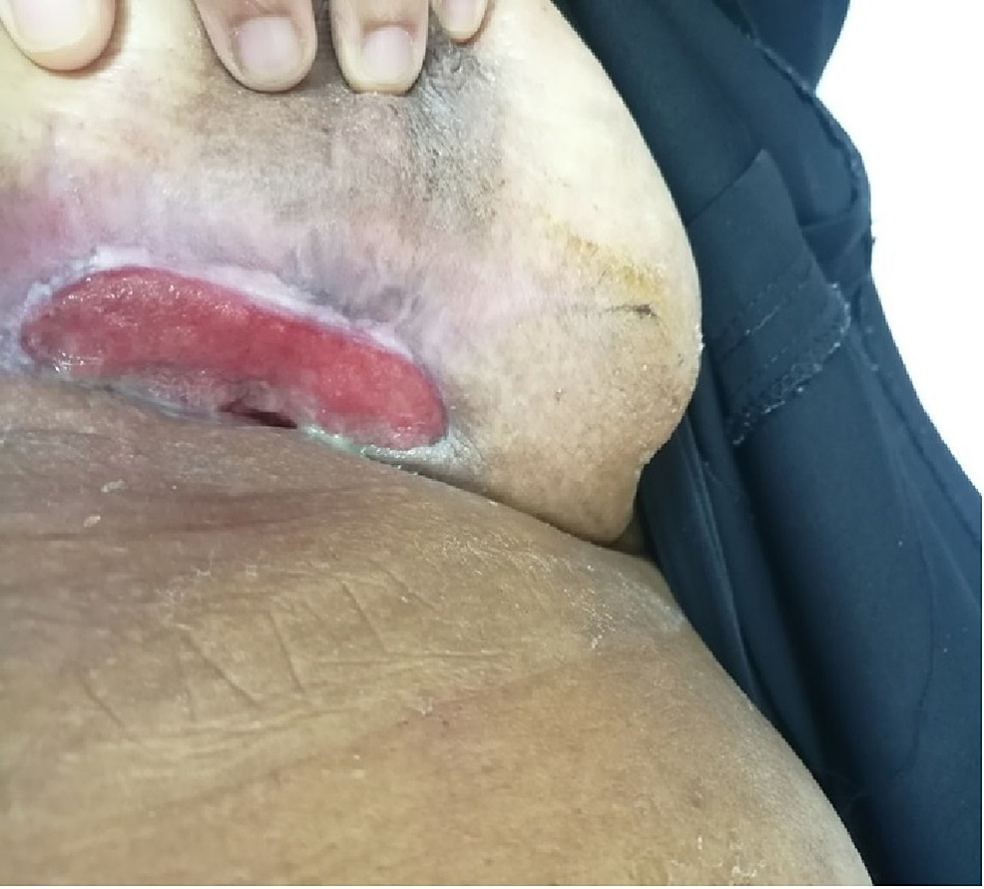
Photo of the breast two months after closure showing good healing progress and healthy tissue

Afterward, the patient was discharged from the hospital two days after the surgery with outpatient clinic follow-up, and no further complication was noted. At the follow-up two months later, the patient was observed to have a completely open granulating wound, with no signs of recurrence or wound infection (Figure 7).

## Discussion

NF of the breast is a very rare entity as NF commonly affects the extremities, trunk, and perineum [5]. It is often misdiagnosed as an abscess or mastitis, similar to our initial diagnosis. Nizami et al. reported the first case of breast NF in 2006, and it involved a female patient who was a known case of hypertension, asthma, and steroid-dependent arthritis, and whose gram-stain culture revealed polymicrobial infection [6]. Another case was reported by Konik et al., of a 53-year-old female who was hypertensive, obese, asthmatic, and a smoker. Furthermore, the patient had an initial abscess that was treated with incision and drainage and was packed accordingly. The authors stated that she presented three days after the abscess treatment with a new presentation of NF [7]. Similarly, our patient was hypertensive, diabetic, and obese but did not report any history of smoking. However, our patient did not report any trauma, surgical incision, or insect bites. ALShareef and ALSaleh have reported the case of a 60-year-old post-menopausal female patient with chronic bilateral inflammation of the breast of six months' duration, which turned into bilateral NF, and the patient underwent a bilateral simple mastectomy. Similar to our case, her history was significant for diabetes and hypertension [5]. In the literature, the reported cases have shown that predisposing risk factors can include hypertension, asthma, diabetes, steroid-dependent treatment, surgical incisions, abscess, mastitis, obesity, and smoking [5-9]. Meanwhile, the increased risk of developing NF of the breast is not yet associated with any particular age group. Singh et al. have reported a case of a 35-year-old female patient who underwent a core-needle biopsy of the breast for a vague lump. A few days later, she presented with a necrotic patch of the breast with severe pus and discharge from the biopsy site. However, she was diabetic with no other reported comorbidities [8].

Previously reported findings have shown mixed culture results from monomicrobial to polymicrobial incidents of NF. Cai et al. studied a total of 40 cases from the literature and reported that around 37.5% were monomicrobial and related to *Streptococcus pyogenes*, whereas mixed organisms were found in 42.5% of the cases [9]. Our patient's culture returned with findings of *Streptococcus anginosus*, which is common in cases of cervical NF following pharyngeal or dental infection [10]. However, to the best of our knowledge, this is the first case of breast NF that was infected by an isolated *Streptococcus anginosus*. *Streptococcus anginosus* is a group of gram-positive streptococci normally colonizing the upper respiratory and digestive and reproductive tracts. However, it is not pathogenic in nature, but rather an opportunistic pathogen inducing any kind of infection in all age groups and immunocompromised patients [11].

Although NF is a very life-threatening condition, early recognition is still difficult as it mimics other inflammatory conditions such as cellulitis and local inflammatory conditions. However, it rapidly progresses to necrotic tissues and can be fatal [12]. Wong et al. developed the Laboratory Risk Indicator for Necrotizing Fasciitis (LRINEC) scoring system to differentiate between NF and other soft tissue infections. The scoring system consists of 13 points with a cut-off value of 6, where patients scoring more than or equal to 6 are associated with a high suspicion for NF. Six variables make up the scoring system: c-reactive protein, total white blood count (WBC), hemoglobin, sodium, creatinine, and glucose levels. Each set of the value range is given a specific score with a maximum score of 13 points. The authors concluded that even though it can help in the early recognition of NF, it is not a definitive tool in diagnosing NF by itself [12]. Incidentally, our patient scored 6, which was an indication of a probable development of NF. Once NF is identified, surgical debridement at the earliest is crucial for the survival of the patient. Lastly, the concurrent COVID-19 infection is hypothesized to be an aggravating factor in this case with further research warranted to study the association in depth.

## Conclusions

NF of the breast is a rare disease. It may be misdiagnosed as cellulitis or an abscess due to delayed cutaneous findings. As a result, the damage might be severe, necessitating a more radical approach toward treatment. Predisposing factors for developing breast NF include hypertension, diabetes, obesity, asthma, and smoking. This case highlights the importance of a multidisciplinary approach to the management of NF involving surgical debridement and chemical debridement to obtain adequate source control and achieve proper reconstruction.
